# The Use of Direct Endoscopic Necrosectomy During Endoscopic Drainage of Walled-Off Pancreatic Necrosis

**DOI:** 10.3390/jcm15051813

**Published:** 2026-02-27

**Authors:** Mateusz Jagielski, Agata Chwarścianek, Jacek Piątkowski, Marek Jackowski

**Affiliations:** Department of General, Gastroenterological and Oncological Surgery, Ludwik Rydygier Regional Hospital in Toruń, 87-100 Toruń, Poland; matjagiel@gmail.com (M.J.); jpiatkowski@wp.pl (J.P.); jackowscy@hotmail.com (M.J.)

**Keywords:** walled-off pancreatic necrosis, necrosectomy, endotherapy

## Abstract

**Introduction**: Endotherapy is an established minimally invasive treatment for pancreatic necrosis. **Aim**: This study aims to evaluate the efficacy and safety of direct endoscopic necrosectomy (DEN) performed during transmural drainage in patients with symptomatic walled-off pancreatic necrosis (WOPN). **Materials and Methods**: A retrospective analysis was conducted of 512 patients with symptomatic WOPN treated endoscopically between 2018 and 2025 at the Department of General, Gastroenterological and Oncological Surgery, Collegium Medicum, Nicolaus Copernicus University in Toruń. In patients qualified for endoscopic necrosectomy, an endoscope was introduced into the necrotic cavity through a previously created transmural (transgastric or transduodenal) fistula, and necrotic tissue was removed using various endoscopic tools. **Results**: All 512 patients underwent transmural endoscopic drainage. Of these, 226/512 (44.14%) patients (61 women, 165 men; mean age 51.8 [20–78] years) were qualified for endoscopic necrosectomy. The mean size of the necrotic collection was 22.9 (10.6–36.6) cm. A transgastric approach was used in 219/226 (96.9%) patients, and a transduodenal approach in 7/226 (3.1%). Active drainage was maintained for a mean of 16 (7–82) days. The mean number of endoscopic procedures was 4.84 (1–24). Complications occurred in 24/226 (10.61%) patients. Mortality was 5.75% (13/226). Clinical success was achieved in 203/226 (89.82%) patients and long-term success in 197/226 (87.17%). **Conclusions**: Direct endoscopic necrosectomy performed during transmural drainage achieved high clinical and long-term success with acceptable morbidity in patients with symptomatic WOPN.

## 1. Introduction

In recent decades, the continuous development of minimally invasive techniques for the treatment of acute necrotizing pancreatitis has been observed, which has been associated with improved outcomes in this group of patients [1]. In the past, open surgical necrosectomy was associated with a high rate of complications and mortality and, for many years, remained the only treatment option for pancreatic necrosis [2]. In the management of acute necrotizing pancreatitis, a step-up approach is currently recommended. This strategy involves the gradual escalation of access to necrotic areas using minimally invasive techniques, including endoscopic transmural approaches, transpapillary (transduodenal) approaches, surgical percutaneous techniques such as sinus tract endoscopy, and the VARD procedure (video-assisted retroperitoneal debridement) [3,4,5].

Endoscopic transmural drainage is currently considered an effective and widely used method for the treatment of symptomatic walled-off pancreatic necrosis (WOPN) [6].

In cases where transmural drainage is ineffective, endoscopic necrosectomy is indicated [7]. It should be emphasized that performing necrosectomy during the drainage of pancreatic necrosis significantly improves treatment efficacy.

In two previous studies by the authors [8] published in 2015 and 2018, the outcomes of patients with walled-off pancreatic necrosis who underwent endoscopic necrosectomy were presented. The 2015 study described an original technique of endoscopic necrosectomy performed under fluoroscopic guidance during transmural drainage of WOPN, which constitutes an alternative to direct endoscopic necrosectomy. In turn, the 2018 study presented the results of treatment in 114 patients with WOPN, confirming the safety and efficacy of this innovative minimally invasive treatment method.

More recently, national and international expert recommendations have further refined the indications, timing, and technical aspects of endoscopic management of pancreatic necrosis [9,10,11,12,13,14]. Optimal timing of the intervention, patient selection, and choice of endoscopic technique remain subjects of ongoing clinical debate.

The technique of direct endoscopic necrosectomy has been modified, and the present study presents the most recent treatment outcomes from a referral center performing direct endoscopic necrosectomy during endoscopic drainage of WOPN. The aim of this publication is to evaluate the efficacy and safety of direct endoscopic necrosectomy performed during transmural drainage in patients with symptomatic walled-off pancreatic necrosis.

## 2. Materials and Methods

### 2.1. Study Design and Ethical Approval

This retrospective single-center study analyzed the outcomes of endoscopic treatment in 512 patients with symptomatic walled-off pancreatic necrosis (WOPN) treated between August 2018 and September 2025 at the Department of General, Gastroenterological and Oncological Surgery, Ludwik Rydygier Collegium Medicum in Bydgoszcz, Nicolaus Copernicus University in Toruń, Poland. The study was approved by the Ethics Committee of the Collegium Medicum of Nicolaus Copernicus University in Toruń, Poland (approval no. KB379.1.1/2018/12) and was conducted in accordance with the Declaration of Helsinki. All patients provided written informed consent for the endoscopic procedures. The diagnosis of acute pancreatitis and classification of local and systemic complications were based on the revised Atlanta classification (2012). Conservative management followed international and Polish guidelines and included intensive fluid therapy, analgesia, and nutritional support. All cases were discussed during interdisciplinary meetings of senior medical staff.

### 2.2. Participants and Eligibility Criteria

#### 2.2.1. Inclusion Criteria for Endoscopic Treatment of WOPN

All patients with clinical symptoms of WOPN secondary to acute or chronic pancreatitis were eligible for endoscopic treatment. Qualification was based on clinical presentation and contrast-enhanced computed tomography (CECT). Endoscopic intervention was delayed until the collection became encapsulated and necrotic material liquefied, forming WOPN, typically ≥4 weeks after the onset of pancreatitis, as confirmed on imaging.

#### 2.2.2. Exclusion Criteria

The following patients were excluded:

- WOPN not related to pancreatic inflammatory disease;
- Asymptomatic WOPN;
- Patients who had previously undergone pancreatic surgery due to acute pancreatitis or its complications;
- Patients who underwent interventional treatment during the early phase (<4 weeks) of acute pancreatitis;
- Patients managed exclusively with non-endoscopic surgical approaches.

A total of 512 patients met the criteria for endoscopic transmural drainage.

#### 2.2.3. Indications for Direct Endoscopic Necrosectomy (DEN)

Among patients undergoing transmural drainage, direct endoscopic necrosectomy (DEN) was performed exclusively in patients who did not demonstrate clinical improvement or who experienced clinical deterioration despite ongoing drainage. Indirect signs of infection were defined as the presence of gas bubbles within the necrotic collection on Contrast-Enhanced Computed Tomography, after the exclusion of gastrointestinal perforation.

### 2.3. Endoscopic Procedure

All procedures were performed in an operating room under general anesthesia with endotracheal intubation and anesthesiology supervision. All procedures were performed by a single experienced endoscopist using carbon dioxide insufflation.

#### 2.3.1. Endoscopic Transmural Drainage

**Figure 1:**
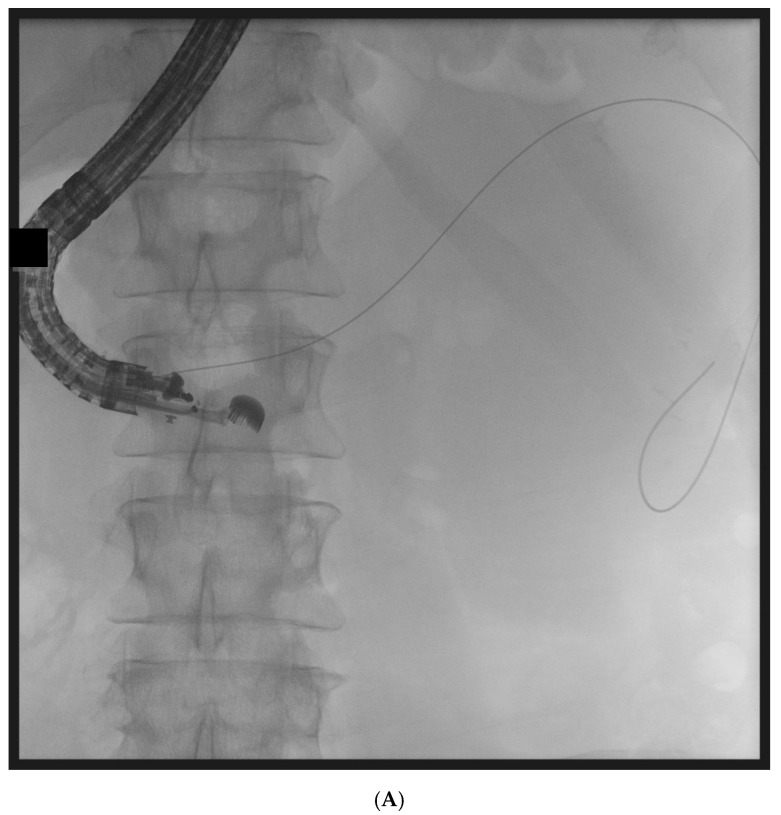

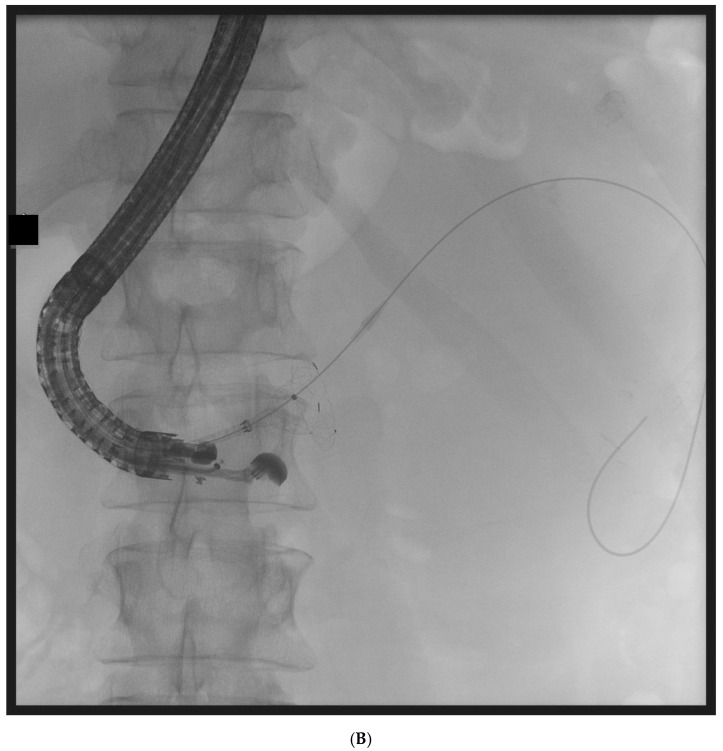

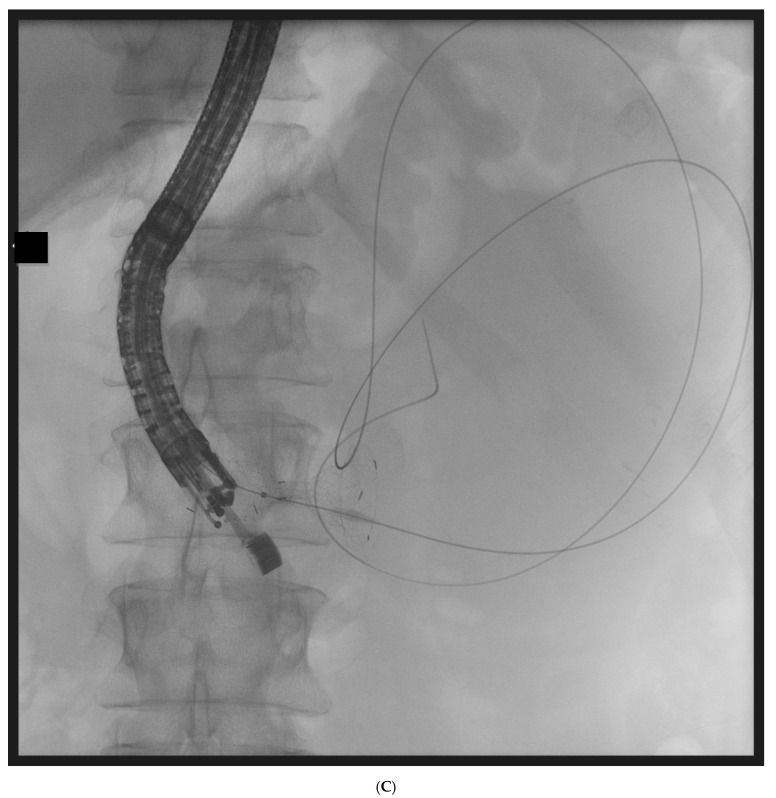

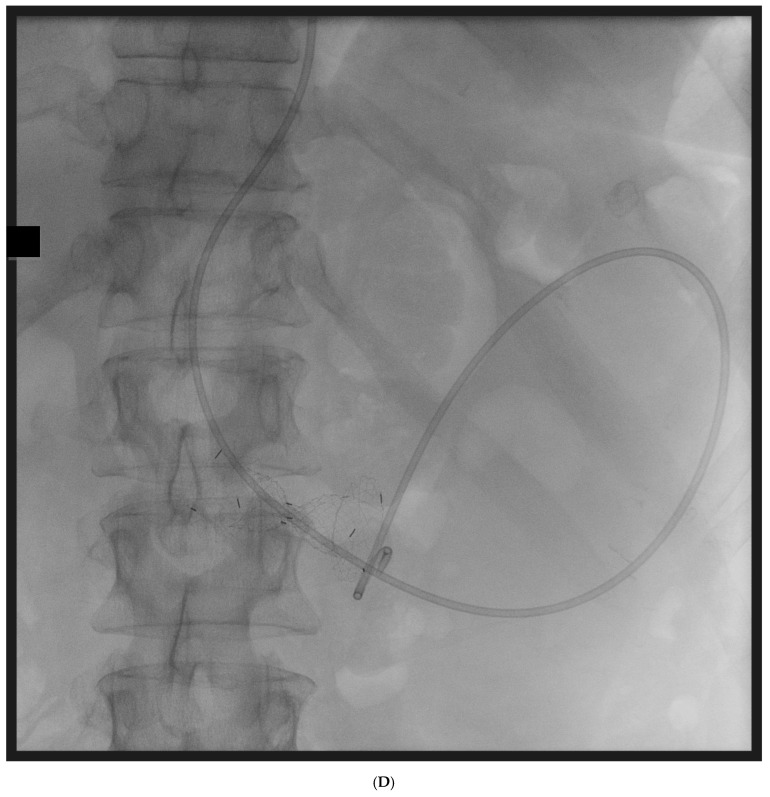
(**A**) Active transmural/transgastric drainage of walled-off pancreatic necrosis. (**B**) Active transmural/transgastric drainage of walled-off pancreatic necrosis. (**C**) Active transmural/transgastric drainage of walled-off pancreatic necrosis. (**D**) Active transmural/transgastric drainage of walled-off pancreatic necrosis.

Transmural drainage was performed under endoscopic ultrasound (EUS) guidance using a linear echoendoscope. After visualization of the collection, transmural puncture (transgastric or transduodenal) was performed using a 19G needle when the distance between the gastrointestinal wall and the collection did not exceed 40 mm. Necrotic material samples were collected for microbiological and laboratory analyses. A guidewire was introduced into the cavity, followed by placement of a 10 Fr cystotome to create the transmural tract. Subsequently, a lumen-apposing metal stent (LAMS) or biflanged metal stent (BFMS) with a diameter ≥ 15 mm (16 or 20 mm) and length of 20–40 mm was deployed. In all patients, additional plastic double-pigtail stents (DPPSs) were inserted through the metal stent to maintain patency and facilitate drainage (Figure 1). A nasocystic drain was placed in all patients after initial transmural drainage. Prophylactic intravenous antibiotics were administered prior to the procedure and continued according to clinical status (third-generation cephalosporin 2 g intravenously and metronidazole 500 mg intravenously three times daily).

#### 2.3.2. Direct Endoscopic Necrosectomy

**Figure 2:**
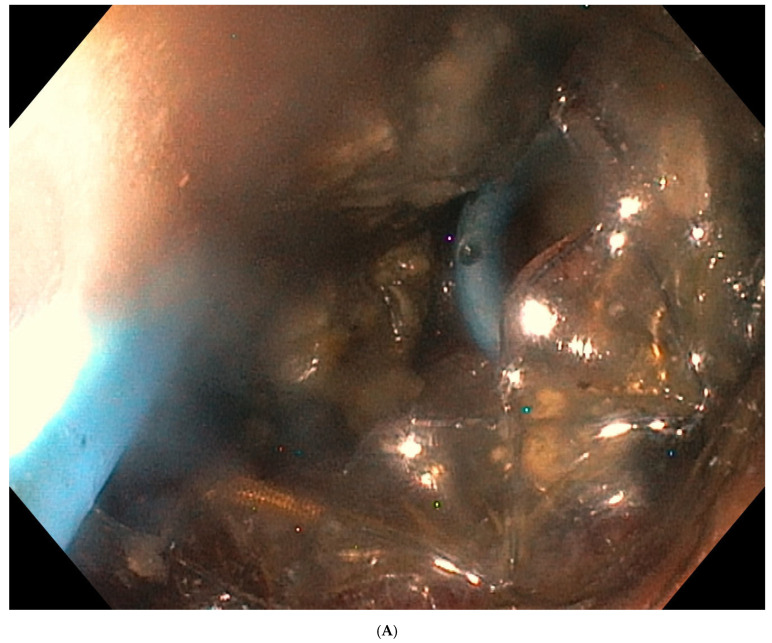

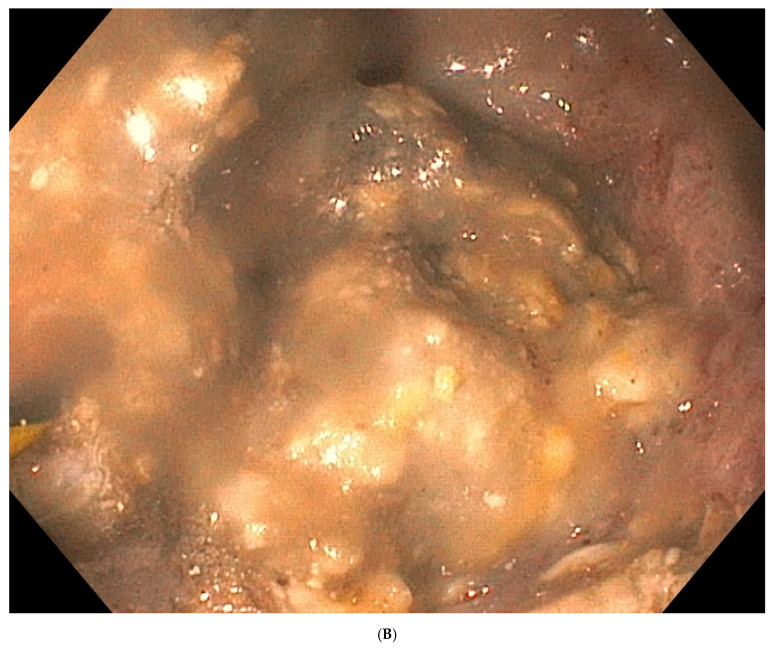
(**A**) Direct endoscopic necrosectomy during transmural drainage of walled-off pancreatic necrosis. (**B**) Direct endoscopic necrosectomy during transmural drainage of walled-off pancreatic necrosis.

DEN was performed through the previously created transmural fistula after removal of the nasocystic drain. A gastroscope was advanced into the necrotic cavity through the LAMS under direct visualization. Mechanical debridement was performed using a Dormia basket (Figure 2). The cavity was repeatedly irrigated with saline solution and aspirated. All procedures were performed with controlled CO_2_ insufflation. DEN sessions were repeated as clinically indicated until satisfactory evacuation of necrotic debris was achieved. After each session, hemostatic powder (Hemospray) was applied within the cavity. A nasocystic drain and/or DPPSs were reinserted as needed to allow for continued evacuation of liquefied debris.

### 2.4. Definitions of Outcomes

Technical success was defined as successful creation of transmural access with proper stent placement and completion of the intended necrosectomy procedure. Clinical success was defined as resolution of collection-related symptoms and total regression of the collection or a reduction in its maximal diameter to <40 mm on follow-up imaging without the need for surgical intervention. Long-term success was defined as the absence of symptoms and total regression or a reduction in the collection to <40 mm at 1 year after discontinuation of active drainage. Recurrence was defined as the reappearance of symptoms or an increase in collection size to >40 mm during follow-up after initial clinical success. The duration of endotherapy was defined as the total period of endoscopic treatment, from removal of the nasocystic drain to removal of the transmural stents. Procedure-related complications included bleeding, perforation, infection, and stent-related adverse events. Complications were graded according to the Clavien–Dindo classification. Early complications were defined as those occurring within 30 days after the procedure and late complications as those occurring thereafter. Bleeding was considered clinically significant if it required blood transfusion, endoscopic intervention, endovascular embolization, or surgical management.

### 2.5. Statistical Analysis

Statistical analyses were performed using STATISTICA software (Version 12.0, StatSoft Inc., Tulsa, OK, USA). Quantitative variables are presented as the mean, standard deviation, median, and range. Qualitative variables are presented as numbers and percentages. Comparisons between groups were performed using appropriate parametric or non-parametric tests depending on data distribution. A *p*-value < 0.05 was considered statistically significant.

## 3. Results

### 3.1. Patient Demographics and Baseline Characteristics

Between August 2018 and September 2025, 512 patients with symptomatic walled-off pancreatic necrosis (WOPN) underwent endoscopic transmural drainage. Of these, 226 patients (44.14%) required direct endoscopic necrosectomy (DEN) and constituted the study cohort. The demographic and baseline characteristics of the study cohort are summarized in Table 1. The study group included 61 women (27.0%) and 165 men (73.0%), with a mean age of 51.8 years (range 20–78). The etiology of necrotizing pancreatitis was alcoholic in 101 patients (44.7%), biliary in 68 patients (30.1%), iatrogenic in 27 patients (11.9%), and idiopathic in 30 patients (13.3%).

### 3.2. Collection Characteristics

The mean size of the necrotic collection was 22.9 cm (range 10.6–36.6 cm). The mean time from onset of acute necrotizing pancreatitis to initiation of endotherapy was 56 days (range 29–129 days). Detailed characteristics of WOPN collections are presented in Table 2.

### 3.3. Procedural Details

A transgastric approach was used in 219 patients (96.9%), and a transduodenal approach in 7 patients (3.1%). Active endoscopic drainage was maintained for a mean of 16 days (range 7–82 days). The mean number of endoscopic procedures per patient was 4.84 (range 1–24). Procedural characteristics are summarized in Table 3.

### 3.4. Clinical Outcomes

Clinical success was achieved in 203 patients (89.82%). Clinical failure occurred in 11 patients (4.87%). Among patients with clinical failure, four died during endotherapy and seven required surgical intervention. Of the surgically treated patients, five died due to the severity of acute pancreatitis. Overall mortality was 13/226 (5.75%). No deaths were directly related to endoscopic complications. It should be noted that overall mortality reflected the severity of acute pancreatitis and systemic complications rather than failure of endoscopic therapy itself; therefore, mortality and clinical failure were not entirely overlapping categories. Treatment outcomes are summarized in Table 4.

During follow-up, recurrence of WOPN occurred in 19 patients (8.4%). Of these, 17 (89.47%) were successfully managed endoscopically, while 2 (10.52%) required surgical treatment. The mean time to recurrence was 82 days (range 61–186 days). Long-term success was achieved in 197 patients (87.17%). The mean follow-up duration was 812 days (range 67–2126 days).

### 3.5. Disconnected Pancreatic Duct Syndrome

Disconnected pancreatic duct syndrome (DPDS) was diagnosed in 49 out of 226 patients (21.7%). The diagnosis was established based on contrast-enhanced computed tomography of the abdomen and fluoroscopic findings during endoscopic retrograde pancreatography. Importantly, all 19 patients who developed recurrence of WOPN were found to have DPDS. In patients with confirmed DPDS, a plastic transmural stent was intentionally left in situ for long-term drainage, which is a frequently used strategy for permanent transmural drainage.

### 3.6. Complications

Procedure-related complications occurred in 24 patients (10.61%). The most common complication was gastrointestinal bleeding (20 patients). Stent migration occurred in three patients and perforation in one patient. The distribution of complications according to the Clavien–Dindo classification is presented in Table 5.

## 4. Discussion

In the present study, we present the outcomes of 226 patients who underwent direct endoscopic necrosectomy (DEN) for symptomatic walled-off pancreatic necrosis (WOPN) in a high-volume tertiary referral center. Clinical success was achieved in 203/226 patients (89.82%), and long-term success was maintained in 197 patients (87.17%) during extended follow-up. Procedure-related complications occurred in 24/226 patients (10.61%), with gastrointestinal bleeding being the predominant adverse event. Mortality in the analyzed cohort was 5.75%, and the mean number of DEN sessions per patient was 4.84. To provide a structured comparison with the existing literature, we summarized the principal outcomes of our cohort alongside landmark studies (GEPARD, Gardner et al., and Yasuda et al.) in Table 6.

The concept of direct endoscopic necrosectomy was first introduced by Seifert et al. in 2000 in a small group of patients with infected WOPN and contraindications to surgical treatment [15]. From the outset, the minimally invasive nature of this technique was recognized as a major advantage, particularly in critically ill patients. The term “direct” refers to the ability to access the necrotic cavity through the gastric or duodenal wall using an endoscope, allowing for direct visualization and mechanical removal of necrotic tissue [16]. Since that initial report, DEN has evolved into a key component of the endoscopic step-up approach for necrotizing pancreatitis. Early single-center experiences further confirmed the feasibility and safety of this technique in selected patients, reporting high technical and clinical success rates with acceptable morbidity [17].

In our center, DEN was performed using conventional tools, primarily a Dormia basket, similarly to the original technique described by Seifert et al. The Dormia basket enables blunt and atraumatic removal of necrotic debris, which is considered advantageous in terms of reducing the risk of bleeding compared with sharp instruments such as forceps. However, its limitations become apparent when managing large or highly septated necrotic collections. The limited capacity of the basket and its reduced ability to grasp loose, irregular fragments necessitate repeated insertion and withdrawal, which prolongs the procedure and may increase cumulative procedural risk [18,19,20,21]. The lack of dedicated devices specifically designed for DEN remains one of the main technical limitations of this method. Recently, novel endoscopic necrosectomy systems, such as the EndoRotor PED System, have been introduced [18,19]. In a prospective multicenter study, the use of this device was associated with a reduced number of DEN sessions and no reported bleeding complications [21]. Despite these promising results, evidence supporting the routine use of such systems remains limited, and long-term comparative data are still lacking. In our center, we do not yet have experience with dedicated necrosectomy devices, and conventional instruments remain the standard of care. Direct endoscopic necrosectomy is a technically demanding procedure and may be associated with severe, potentially life-threatening complications.

Gastrointestinal bleeding is consistently reported as the most frequent adverse event. In our cohort, bleeding accounted for 83% of all complications and was successfully managed conservatively in most cases. Only a small proportion of patients required endoscopic or surgical intervention. These findings are consistent with previous reports and underscore the importance of careful patient selection and procedural expertise. When compared with the available literature, the outcomes achieved in our study are comparable to those reported in major multicenter series. In the GEPARD study, DEN performed in 93 patients resulted in therapeutic success in 81%, with a complication rate of 26% and a mean number of 6.2 procedures per patient [16]. The reported complications in the GEPARD cohort included bleeding, perforation, infection, and stent-related adverse events, reflecting a mixed complication profile. Gardner et al. reported therapeutic success in 91% of 104 patients, with a complication rate of 14% [22]. Yasuda et al. reported outcomes of endoscopic necrosectomy in a retrospective multicenter cohort of 57 patients with infected WOPN, achieving clinical success in 75% of cases, with a complication rate of 33% and a mortality rate of 11% [23].

As summarized in Table 6, our clinical success, complication profile, mortality, and procedural burden fall within the range reported in these landmark studies, further supporting the effectiveness of DEN when performed in experienced centers. The timing of intervention remains a subject of ongoing debate. In our study, DEN was initiated after maturation of WOPN, typically more than four weeks after the onset of acute pancreatitis. This strategy reflects long-standing clinical practice aimed at reducing complications associated with early intervention. Randomized trials and observational studies have failed to demonstrate a clear benefit of early (<4 weeks) intervention and have, in some cases, reported increased mortality and a higher need for rescue surgery [24,25]. The recent POINTER trial comparing immediate versus postponed drainage also did not demonstrate the superiority of early intervention and reported a higher number of interventions in the immediate-drainage group, further supporting a delayed strategy after encapsulation of necrotic collections. Our results are consistent with these observations and support delayed intervention after encapsulation of necrotic collections.

During long-term follow-up, recurrence of WOPN was observed in 19 patients. Importantly, seventeen of these recurrences were successfully treated endoscopically, while only two patients required surgical management. These findings emphasize the durability of the endoscopic step-up approach and the feasibility of repeat endoscopic intervention in selected cases. Recurrence in our cohort was closely associated with disconnected pancreatic duct syndrome (DPDS). In 49 patients (21.7%), DPDS was diagnosed based on contrast-enhanced computed tomography and fluoroscopic findings during endoscopic retrograde pancreatography. Importantly, all patients who developed recurrence of WOPN were found to have DPDS. These findings strongly suggest that disruption of the main pancreatic duct was the principal mechanism underlying recurrence in our cohort. Disruption of the main pancreatic duct is a frequent consequence of necrotizing pancreatitis, with reported prevalence ranging from 30% to 50% [26,27]. DPDS should be distinguished from simple duct disruption without clinical consequences; in DPDS, pancreatic secretions from the disconnected segment are not drained physiologically into the duodenum, leading to recurrent fluid collections, persistent fistulae, or other complications [26,27,28,29].

The diagnosis of DPDS is primarily based on cross-sectional imaging modalities, including contrast-enhanced CT, MRI/MRCP, EUS, and ERCP. Accurate identification of ductal disruption is crucial, as failure to restore physiological drainage may predispose patients to recurrence of peripancreatic collections. Various management strategies have been described, including long-term transmural stenting and surgical approaches [30,31,32,33]. In clinical practice, endoscopic transpapillary stenting may be considered in selected cases; however, successful bridging of the disrupted duct is often technically challenging and not always feasible, particularly in cases of complete ductal disconnection. In patients with confirmed DPDS, a plastic transmural stent was intentionally left in situ for long-term drainage, which is a frequently used strategy of permanent transmural drainage. This approach aimed to maintain continuous internal drainage of pancreatic secretions from the disconnected segment and reduce the risk of recurrent fluid collections.

In our experience, the inability to achieve or maintain physiological pancreatic duct drainage likely contributed to recurrence in a subset of patients. These observations highlight the importance of careful evaluation of ductal anatomy during follow-up and suggest that long-term management of WOPN should include consideration of underlying ductal disruption.

The present study has several limitations. Its retrospective, single-center design may limit generalizability. Nevertheless, the strengths of this study include a large cohort of patients treated with DEN and an extended follow-up period, allowing for reliable assessment of long-term outcomes and recurrence.

**Table 6:** Comparison of outcomes of direct endoscopic necrosectomy (DEN) with major published studies.

Parameter	Present Study (2018–2025)	GEPARD (Seifert et al., 2009 [16])	Gardner et al., 2011 [22]	Yasuda et al., 2013 [23]
Study design	Single-center	Multicenter	Multicenter	Multicenter
Number of patients (*n*)	226	93	104	57
Mean age (years)	51.8	57	NR	NR
Mean time to intervention (days)	56	43	63	NR
Technical success (%)	89.82	NR	91	75
Clinical success (%)	87.17 (long-term)	80 (initial)	91.3	75
Overall complication rate (%)	10.61	26	~14	33
Most common complication	Bleeding	Mixed	NR	NR
Mortality (%)	5.75	7.5	5.7	11
Mean number of DEN sessions	4.84	~6	~3	5
Recurrence rate (%)	8.4 (19/226)	10–14	NR	~7
Mean follow-up	812 days	43 months	4.1 months	27 months

NR—not reported in the original publication.

## 5. Conclusions

Direct endoscopic necrosectomy performed as part of a transmural step-up approach is an effective minimally invasive treatment for symptomatic walled-off pancreatic necrosis. In our large single-center cohort of 226 patients, DEN achieved clinical success in 89.82% of cases, with long-term success maintained in 87.17% during extended follow-up. Procedure-related complications occurred in 10.61% of patients, with gastrointestinal bleeding being the most common adverse event.

Our findings confirm that DEN significantly improves the outcomes of endoscopic management of pancreatic necrosis when applied after adequate maturation of the collection. The durability of treatment is further supported by the high rate of successful endoscopic management of recurrences (17/19 cases), highlighting the feasibility of repeat endoscopic intervention within a structured step-up strategy.

The timing of DEN remains an important clinical consideration. In our cohort, interventions were performed after encapsulation of WOPN (>4 weeks), and this delayed strategy was associated with favorable outcomes. These data support postponing intervention until adequate wall formation whenever clinically feasible.

The lack of dedicated devices specifically designed for necrosectomy remains a technical limitation of the procedure. Although emerging systems such as powered endoscopic debridement devices appear promising, currently available evidence is insufficient to allow us to draw definitive conclusions regarding their superiority or safety.

The strengths of the present study include the large number of patients undergoing DEN and the long follow-up period, which—based on the available literature—represents one of the largest single-center experiences with this minimally invasive technique.

## Figures and Tables

**Table 1:** Demographic and baseline characteristics (*n* = 226).

Characteristic	Value
Age, mean (range), years	51.8 (20–78)
Male sex, *n* (%)	165 (73.0%)
Female sex, *n* (%)	61 (27.0%)
Alcoholic etiology, *n* (%)	101 (44.7%)
Biliary etiology, *n* (%)	68 (30.1%)
Iatrogenic etiology, *n* (%)	27 (11.9%)
Idiopathic etiology, *n* (%)	30 (13.3%)

**Table 2:** Characteristics of WOPN collections.

Characteristic	Value
Collection size, mean (range), cm	22.9 (10.6–36.6)
Time to intervention, mean (range), days	56 (29–129)

**Table 3:** Procedural characteristics.

Characteristic	Value
Transgastric approach, *n* (%)	219 (96.9%)
Transduodenal approach, *n* (%)	7 (3.1%)
Duration of drainage, mean (range), days	16 (7–82)
Number of sessions, mean (range)	4.84 (1–24)

**Table 4:** Treatment outcomes.

Outcome	Value
Clinical success, *n* (%)	203 (89.82%)
Long-term success, *n* (%)	197 (87.17%)
Recurrence, *n* (%)	19 (8.4%)
Overall mortality, *n* (%)	13 (5.75%)
Disconnected pancreatic duct syndrome, *n* (%)	49 (21.7%)

**Table 5:** Procedure-related complications according to the Clavien–Dindo classification.

Clavien–Dindo Grade	Complication	*n* (%)
II	Bleeding requiring transfusion	9 (3.98%)
IIIa	Endoscopic hemostasis	6 (2.65%)
IIIa	Endovascular embolization	3 (1.33%)
IIIa	Endoscopic stent removal (migration)	3 (1.33%)
IIIb	Surgical management of bleeding	2 (0.88%)
IIIb	Surgical management of perforation	1 (0.44%)
IV	—	0

## Data Availability

Data supporting the findings of this study are available from the corresponding author upon reasonable request.

## Abbreviations

The following abbreviations are used in this manuscript:

VARD	video-assisted retroperitoneal debridement
DEN	direct endoscopic necrosectomy
MARPN	minimal access retroperitoneal pancreatic necrosectomy
MIRPN	minimally invasive retroperitoneal pancreatic necrosectomy
DPDS	disconnected pancreatic duct syndrome
